# Cumulative costs of severe traffic injuries in Finland: a 2-year retrospective observational study of 252 patients

**DOI:** 10.1038/s41598-024-61184-2

**Published:** 2024-05-14

**Authors:** Antti Riuttanen, Erkka Karjalainen, Jarkko Jokihaara, Tuomas T. Huttunen, Ville M. Mattila

**Affiliations:** 1 Department of Orthopedics, Tampere University Hospital, Tampere, Finland; 2 Department of Orthopedics, Faculty of Medicine and Health Technology, Tampere University, and Tampere University Hospital, Tampere, Finland; 3 Department of Anesthesia and Intensive Care Medicine, Heart Hospital, Tampere University Hospital, Tampere, Finland

**Keywords:** Health care economics, Health policy, Public health

## Abstract

Road traffic injuries cause considerable financial strain on health care systems worldwide. We retrospectively analyzed injury-related costs of 252 severely injured (New Injury Severity Score, NISS ≥ 16) patients treated at Tampere University Hospital (TAUH) between 2013 and 2017, with 2-year follow-up. The costs were divided into direct treatment, indirect costs, and other costs. We analyzed various injury- and patient-related factors with costs. The total costs during the 2-year study period were 20 million euros. Median cost was 41,202 euros (Q1 23,409 euros, Q3 97,726 euros), ranging from 2,753 euros to 549,787 euros. The majority of costs (69.1%) were direct treatment costs, followed by indirect costs (28.4%). Other costs were small (5.4%). Treatment costs increased with the severity of the injury or when the injury affected the lower extremities or the face. Indirect costs were higher in working age patients and in patients with a higher level of education. The relative proportions of direct and indirect costs were constant regardless of the amount of the total costs. The largest share of costs was caused by a relatively small proportion of high-cost patients during the 1st year after injury. Combined, this makes planning of resource use challenging and calls for further studies to further identify factors for highest costs.

## Introduction

Traffic injuries impose a considerable financial strain on societies. According to the WHO, approximately 1.3 million people die from injuries sustained in road traffic injuries every year. Moreover, road traffic injuries are estimated to account for 3% of gross domestic product in many countries^1^. Nevertheless, there is a scarcity of published studies on the costs of injuries sustained in traffic injuries on both a national and international level.

In 2020, a large survey of 32 European countries reported estimated average costs ranging from 28,205 to 975,074 euros for each severe road traffic injury^2^. However, as both the definition of severe injury and the way in which the costs were calculated varied considerably between countries, the usability of these cost estimations is limited. For Finland, the study used the willingness to pay (WTP) model, where the goal is to give the amount of money that an individual injured patient would be willing to pay to prevent the accident. According to the study, the estimated cost of a single preventable serious traffic injury in Finland was 671,383 euros. However, the amount given by WTP method is influenced by individual preferences and can therefore exceed the actual costs of an accident^3^. Therefore, our primary aim was to evaluate the actual costs per patient of severe traffic injury over a 2-year period in Finland. In addition, we also investigated how costs of injury originate and divided them into direct, indirect, or other costs. We hypothesized that certain patient-related factors, such as age, physical health, level of education, and injury-related factors, including injury severity, injury pattern, mechanism of injury, are associated with these costs.

## Material and methods

### Study design, patients, and time horizon

**Figure 1:**
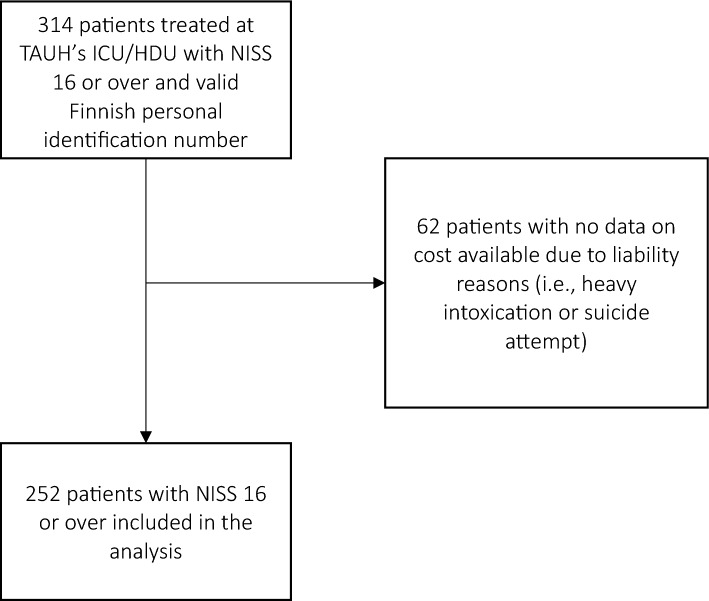
Flowchart of patient selection.

This retrospective observational cohort study included patients who were severely injured in traffic accidents and treated at the intensive care unit (ICU) or high dependency unit (HDU) of Tampere University Hospital (TAUH) between 2013 and 2017. TAUH serves as a tertiary and highly specialized trauma care center for the surrounding 3 hospital districts and has a catchment population of approximately 900,000 inhabitants. Finland has a universal health care system, which covers the whole population, and all major trauma is treated in public hospitals. All patients who were admitted to the ICU or HDU during the study period after sustaining traffic-related injuries were retrospectively screened for study eligibility. The following inclusion criteria were used: patient treated at TAUH’s ICU or HDU due to traffic-related injury, New Injury Severity Score (NISS) ≥ 16, and possession of a valid Finnish personal identification number, which enables comprehensive follow-up. Exclusion criteria were missing cost data due to liability reasons (i.e., heavy intoxication or suicide attempt) (Fig. 1). Injury severity was scored by an anatomically based Abbreviated Injury Scale (AIS)^4^ that classifies each injury to 1 of 9 body regions (head, face, neck, thorax, abdomen, spine, upper extremity, lower extremity and pelvis, and unspecified) on a six-point scale. Based on the AIS scores, New Injury Severity Scores (NISS) were calculated^5^ and a severe injury was defined by a NISS score of at least 16, which is the universally accepted definition of severe trauma in studies concerning traumatology^6^. Therefore, the study cohort represents typical patients with severe traffic injury who are treated at a tertiary trauma center. All patients in this study also fulfilled the criteria for a Maximum Abbreviated Injury Score of 3 or higher (MAIS ≥ 3), which is the definition of severe injury suggested by the European Commission^7^. Physical health was assessed by the ASA (American Society of Anesthesiologists) Physical Status Classification System, which is commonly used in ICUs to assess the pre-anesthesia medical co-morbidities of a patient. Two-year mortality and cause of death data were obtained from Statistics Finland^8^.

The costs of treatment were obtained from the Finnish Motor Insurers’ Centre (LVK)^9^. In accordance with the Act governing motor liability insurance, all vehicles that have Finland as their permanent location must have motor liability insurance^10^. The act also obligates companies granting motor liability insurance to be members of the Finnish Motor Insurers' Centre. As a result, all costs which are paid by the insurance companies due to traffic injuries are available from the LVK (only gross negligence limits liability, i.e., when injury is caused by attempted suicide or heavy intoxication (i.e., blood alcohol content over 1.5 g/L)). In the present study, we included costs that occurred within 2 years of the accident.

The costs were classified in 3 categories: direct, indirect, and other costs (Table 1). Direct costs included those costs resulting from inpatient treatment at our hospital, outpatient visits to the hospital or health care center, cost of rehabilitation, and reimbursement of medicine expenses. Indirect costs included compensation for loss of income, compensation for permanent functional limitation, and disability pension. Other costs included compensation for temporary pain or other harm, and funeral expenses. The 2-year cumulative costs for each cost type were defined by comparing the cumulative costs of the 1st and 2nd year with the cumulative costs of the 2nd and 3rd year for each individual participant and the highest one was then chosen. This method was chosen because the costs for those patients who were injured at the end of year were recorded for the following year (for example costs for injury in December 2014 was recorded for the year 2015).

**Table 1:** Classification of costs.

Direct costs	Indirect costs	Other costs
Treatment provided in hospital, polyclinic visits to hospital or health care center	Compensation for loss of income	Compensation for pain or other temporary harm
Rehabilitation	Compensation for functional limitation	Funeral costs
Reimbursement of medicine expenses	Disability pension	

The causes of injuries were classified with the injury-related 10th revision of the International Classification of Diseases and Related Health Problems (ICD-10) into four categories: 1) car includes all motor vehicle collisions, such as crashes and driving off the road (V49), 2) motorbike includes all motorbike crashes (V28, V29), 3) pedestrian/bicycle auto accidents include injury caused to pedestrians (V01-V09) or to pedal cyclists (V10-V19), and 4) other injuries include miscellaneous injuries, such as collisions with trains (V81) and all-terrain vehicle accidents (V86).

Information on the education level of each individual patient was obtained from Statistics Finland^11^, which is a national public authority for statistical data. Education level was assessed because it may influence, for example, compensation for lost income or ability to return to work after injury. Level of education was defined as low, medium, or high. Those with a low level of education had had at most 9 years of education (elementary school or less), those with a medium level of education had 11 to 12 years of education (including matriculation examination, vocational qualifications attained in 1–3 years, and further vocational qualifications), and those with a high level of education had had more than 12 years of education (for example, polytechnic degrees, lower and upper-level university degrees, and doctoral education).

### Statistics

Statistical analysis was performed with R Studio 4.0.3 (R Foundation for Statistical Computing, Vienna, Austria) with ggplot2, dplyr, xlsx, rehape2, and zoo extra packages. Response variables were 2-year cumulative total, indirect, direct, and other costs. Independent variables were grouped age, sex, ASA, level of education, mechanism of injury, and NISS. Injury pattern variables were grouped into head, neck, thorax, abdomen, spine, upper extremity, lower extremity, and unspecified. Minor (AIS1) injuries, such as cuts and bruises, were excluded from the analysis. Each independent variable was categorical and response variables were not normally distributed continuous variables, so nonparametric Kruskall-Wallis test was used to compare groups. A Kruskall-Wallis test was performed separately for each response variable. A p-value less than 0.05 was considered statistically significant.

### Ethics, funding, and potential conflicts of interest

The study was approved by Pirkanmaa Hospital District. Additional study permits were obtained from all register holders (Pirkanmaa Hospital District, Finnish Motor Insurers’ Centre, Statistics Finland). In Finland, full ethical board review or informed consent is not required for register-based studies (Medical Research Act, 488/1999). This study was financially supported in part by the Competitive State Research Financing of the Expert Responsibility area of Tampere University Hospital. The authors have no conflict of interests to declare.

## Results

During the study period, a total of 314 traffic-injury patients with NISS ≥ 16 were treated at TAUH. Of these, detailed costs were available for 252 patients. The costs were not available due to liability issues for 62 patients who were either heavily intoxicated (i.e., blood alcohol content over 1.5 g/L) or injury was caused by attempted suicide (Fig. 1). The characteristics of the included patients are summarized in Table 2. Mean age of patients was 43.3 years (SD 22.4). 11 patients (4.4%) died within a few days of sustaining the injury. There were also 4 additional non-injury-related deaths close to the end of 2-year follow-up period and 8 non-injury-related deaths after the 2-year follow-up period.

**Table 2:** Demographics of traffic injury patients treated at TAUH's ICU* or HDU† with NISS‡ > 16 (2013–2017).

	n = 252	%
Age		
< 18 years	43	17
18–64 years	150	60
65 and over	59	23
Sex		
Female	83	33
Male	169	67
ASA§		
1–2	205	81
3–4	47	19
Injury severity scores, median (interquartile range)		
NISS‡	22 (12)	
Level of education		
Low	87	35
Medium	109	43
High	56	22
Mechanism of injury		
Car	103	41
Motorbike	70	28
Pedestrian/bicycle auto accident	69	27
Other	10	4.0
Injury pattern (AIS** minimum 2)		
Head	119	47
Face	36	14
Neck	10	4.0
Thorax	153	61
Abdomen	65	26
Spine	87	35
Upper extremity	98	39
Lower extremity and pelvis	113	45
Unspecified	15	6.0
Length of stay in ICU†, median (interquartile range)	1.3 (3.0)	

* Intensive Care Unit.

† High Dependency Unit.

‡ New Injury Severity Score.

§American Society of Anesthesiologist (ASA) physical status classification system.

** Abbreviated Injury Scale.

**Figure 2:**
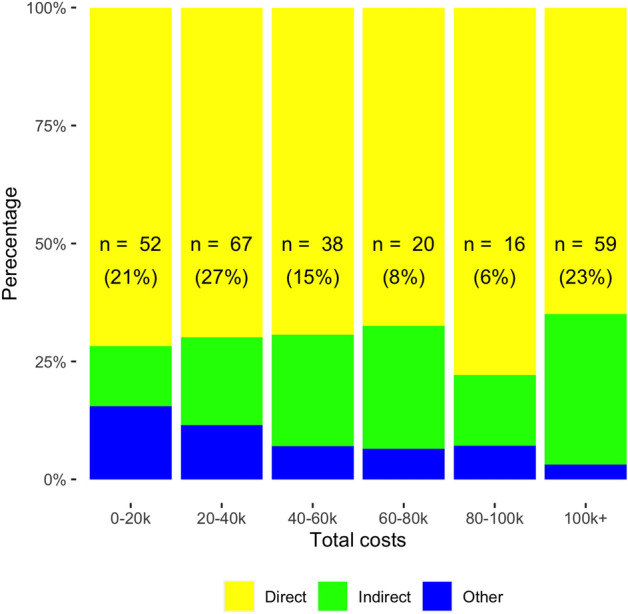
Relative proportions of different cost categories during the two-year follow-up of 252 patients.

**Figure 3:**
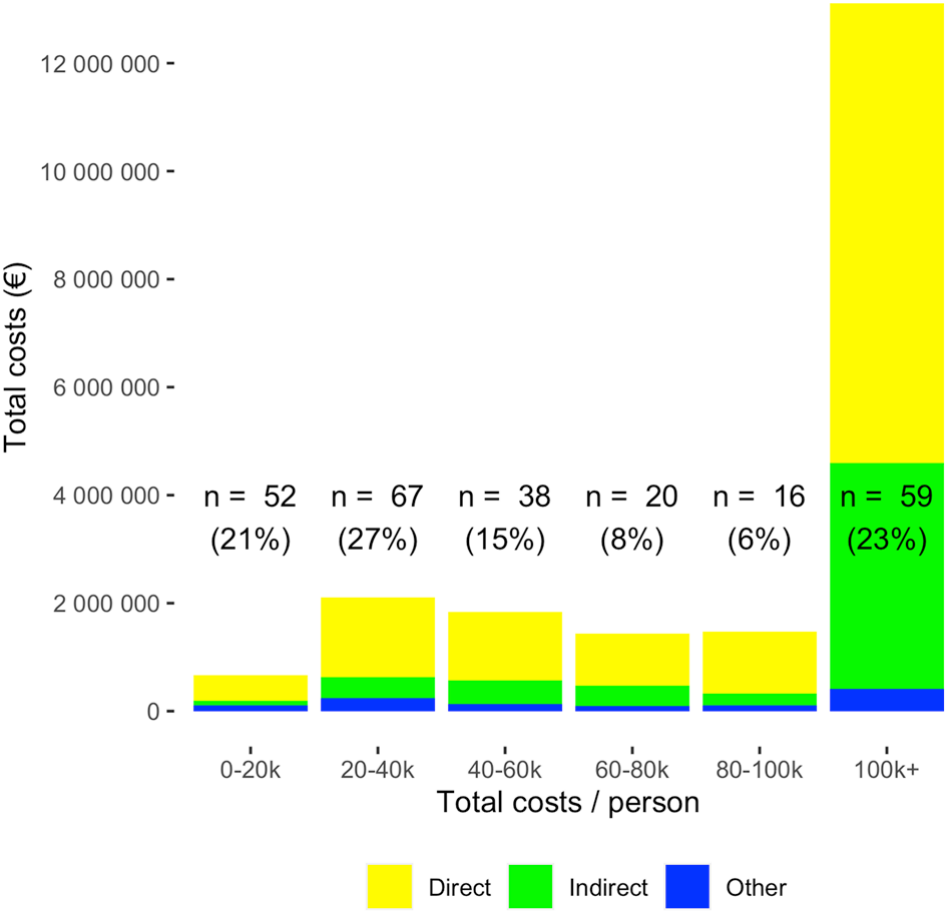
Absolute cumulative two-year costs of different cost categories in 252 patients.

**Figure 4:**
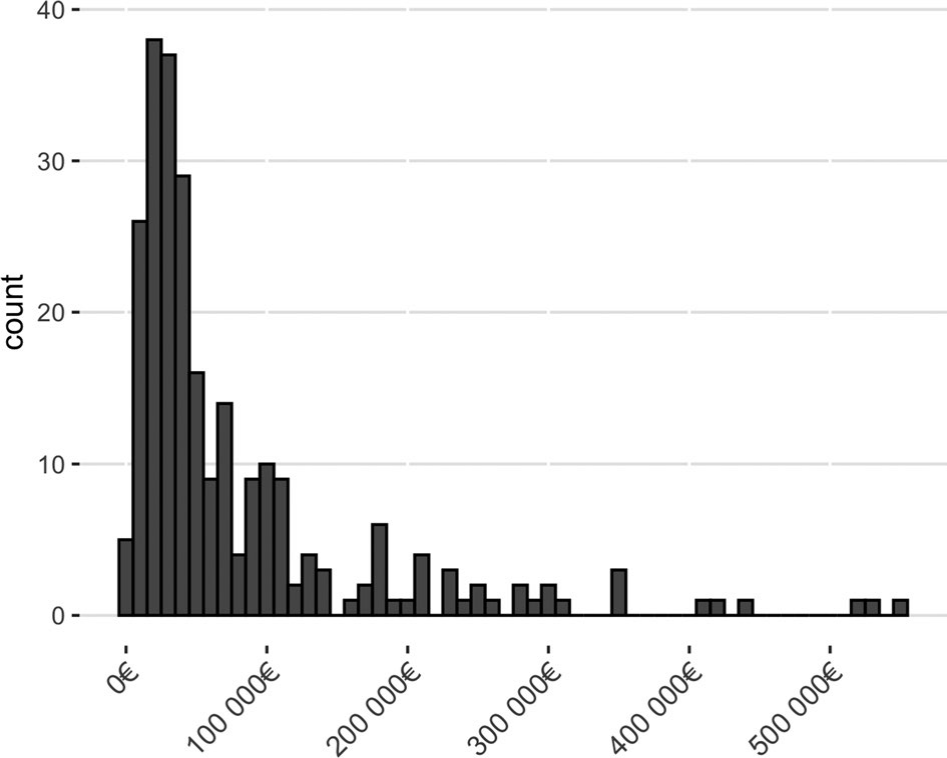
Distribution of the total cost for the 252 patients.

**Figure 5:**
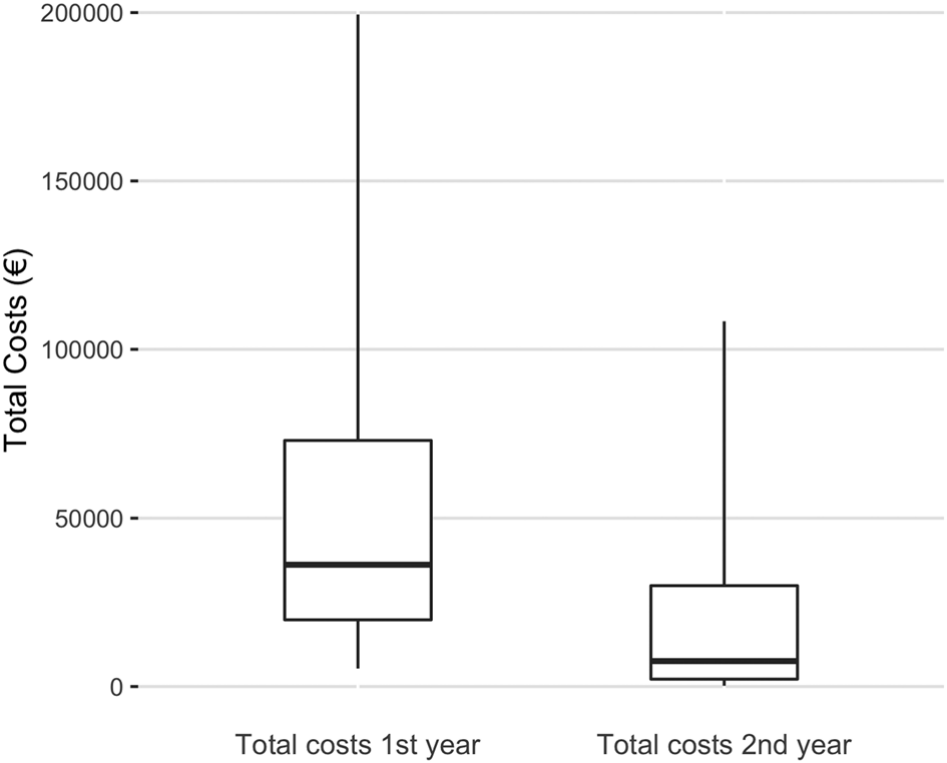
Median total costs of each patient during the 1st and 2nd year of follow-up after the injury. A line presents the median value, a box the interquartile range, and whiskers the range of total cost per patient.

During the 2-year follow-up period, the total cost of the 252 severe traffic injuries was 20 million euros. The majority of the costs (69.1%, 13.8 million euros) were attributed to direct treatment costs followed by indirect costs (28.4%, 5.7 million euros). Other costs included compensation for pain or other temporary harm, and funeral costs (5.4%, 1.1 million euros). The median total cost of 252 patients was 41,202 euros (Q1 23,409 euros, Q3 97,726 euros), ranging from 2,753 euros to 549,787 euros. The proportions of different costs (direct, indirect, and other) are presented in Figs. 2 and 3. The majority of costs (63.8%, 12.8 million euros) was caused by patients whose total costs exceeded 100,000 euros (23.4%, 59/252 patients). The distribution of total costs per patient is presented in Fig. 4. The distribution of total costs during the 1st and 2nd year after injury is presented in Fig. 5. The majority of costs (77.7%, 15.5 million euros) occurred during the 1st year. For the 11 patients who died during the 2-year follow-up period, the median total costs were 29,667 euros (Q1 21,083 euros, Q3 73,608 euros), and the median direct costs were similar when compared to the whole study population (27,501 euros vs 29,641 euros).

Patient- and injury-related factors and their association with costs (direct, indirect, and other) are summarized in Tables 3 and 4, respectively. Total costs were higher in patients with a higher injury severity score and in those patients who had suffered an injury to the lower extremities and pelvis. Direct costs were also higher in patients with higher injury severity and injury to the face or lower extremity, whereas sex, ASA, level of education, or injury mechanism did not have a statistically significant effect on total or direct costs. However, patients aged 18 to 64 years, patients with lower ASA (1–2), and a medium or high level of education had statistically higher indirect costs. Other costs were significantly higher in patients with injuries to the face, neck, or lower extremities.

**Table 3:** Patient-related factors and distribution of costs (median, interquartile range).

	Total costs	*p*-value (*)	Direct costs	*p*-value (*)	Indirect costs	*p*-value (*)	Other Costs	*p*-value (*)
Age								
< 18 years	28,204 (58,798)	0.049	22,017 (49,660)	0.52	1256 (7482)	< 0.001	3500 (1800)	0.13
18–64 years	44,190 (83,961)		29,485 (45,022)		14,008 (39,176)		3800 (2500)	
65 and over	39,258 (56,704)		31,880 (44,961)		1320 (6184)		3700 (2250)	
Sex								
Female	34,583 (51,504)	0.16	25,113 (38,229)	0.19	6235 (18,986)	0.63	3800 (2200)	0.92
Male	45,631 (76,453)		33,814 (50,576)		7202 (27,446)		3800 (2600)	
ASA†								
1–2	42,182 (80,017)	0.39	27,820 (53,036)	0.58	8141 (27,026)	< 0.01	3800 (2500)	0.37
3–4	39,097 (42,414)		31,880 (36,158)		739 (8846)		3700 (2292)	
Level of education								
Low	39,097 (50,425)	0.22	28,141 (44,744)	0.76	2362 (8552)	< 0.001	3500 (1850)	0.44
Medium	46,792 (77,513)		29,660 (44,726)		10,394 (30,238)		3800 (2400)	
High	41,761 (87,568)		34,126 (78,309)		8485 (37,791)		3800 (3143)	

* Kruskall-Wallis test.

† American Society of Anesthesiologist (ASA) physical status classification system.

**Table 4:** Injury-related factors and distribution of costs (median, interquartile range).

	Total costs	*p*-value (*)	Direct costs	*p*-value (*)	Indirect costs	*p*-value (*)	Other Costs	*p*-value (*)
New Injury Severity Score, NISS								
16–24	36,880 (49,572)	< 0.001	23,787 (32,680)	< 0.001	6710 (32,680)	0.97	3600 (1800)	0.0040
25–40	46,796 (75,605)		35,245 (48,891)		6879 (48,891)		3800 (2691)	
40–75	99,925 (178,592)		72,224 (106,560)		5474 (22,959)		6000 (5450)	
Mechanism of injury								
Car	41,341 (70,998)	0.42	29,622 (49,396)	0.54	5474 (25,387)	0.0025	3800 (2500)	0.70
Motorbike	42,305 (84,482)		26,567 (49,033)		8521 (36,362)		3750 (2250)	
Pedestrian/bicycle auto accident	34,583 (61,449)		30,444 (44,058)		5112 (17,425)		3600 (2500)	
Other	80,709 (107,132)		51,162 (64,559)		19,954 (50,100)		4200 (1075)	
Injury pattern (AIS† minimum 2)								
Head								
Yes	39,415 (75,132)	0.51	30,352 (46,366)	0.31	7879 (29,220)	0.19	3800 (2400)	0.07
No	41,118 (67,560)		28,743 (49,702)		5897 (17,781)		3700 (2400)	
Face								
Yes	54,768 (67,502)	0.11	46,010 (67,502)	0.02	7595 (17,181)	0.99	4200 (2300)	0.02
No	40,116 (68,500)		27,816 (46,543)		6321 (24,490)		3700 (2300)	
Neck								
Yes	43,954 (44,189)	0.53	35,640 (34,723)	0.30	6650 (12,849)	0.43	4400 (2725)	0.02
No	41,118 (74,812)		28,729 (48,109)		6515 (24,307)		3800 (2600)	
Thorax								
Yes	42,718 (73,370)	0.094	31,044 (48,737)	0.087	8465 (27,014)	0.060	3800 (2400)	0.24
No	39,907 (69,900)		25,782 (47,806)		4686 (21,458)		3600 (2700)	
Abdomen								
Yes	38,415 (51,583)	0.34	25,604 (43,561)	0.54	5711 (16,146)	0.69	3800 (2462)	0.21
No	44,948 (76,349)		30,613 (48,941)		7401 (27,202)		3550 (2625)	
Spine								
Yes	42,112 (77,825)	0.69	30,352 (44,640)	0.67	7986 (24,253)	0.94	3800 (2325)	0.92
No	40,102 (61,646)		29,622 (51,452)		6296 (23,329)		3800 (2600)	
Upper extremity								
Yes	42,428 (65,645)	0.60	31,100 (49,428)	0.53	5679 (21,735)	0.99	3800 (2100)	0.077
No	40,034 (77,309)		28,115 (47,235)		7587 (24,497)		3700 (2434)	
Lower extremity and pelvis								
Yes	47,829 (77,349)	0.0011	34,183 (56,823)	< 0.001	4498 (20,346)	0.54	3800 (2025)	0.013
No	32,801 (16,965)		22,175 (31,693)		3495 (18,954)		3550 (2392)	
Unspecified								
Yes	56,207 (83,441)	0.67	46,010 (73,505)	0.33	5640 (9876)	0.61	4200 (1600)	0.46
No	40,455 (83,441)		28,715 (45,956)		7202 (24,468)		3800 (2500)	

* Kruskall-Wallis test.

† Abbreviated Injury Scale.

## Discussion

We investigated the costs of severe traffic injuries over a 2-year period in Finland and found that more than 2/3 of costs consisted of direct treatment costs, 3/4 of total costs were accumulated during the 1st year after sustaining the injury. Majority of total costs total costs were generated by less than 1/4 of patients. The relative proportions of direct and indirect costs were constant regardless of the amount of the total costs.

In this study we noticed few factors that associated with higher costs. The amount of direct treatment costs increased with the severity of the injury and high costs were associated particularly with injuries in the lower limbs/pelvis or the facial area. The driving factor for costs may be that high-energy injuries of the lower limbs or pelvis often require surgical treatment and are prone to post-operative complications such as infections^12^. In facial injuries, the cause for high cost is most likely similar, but in addition, injuries to the facial area are often accompanied by intracranial injuries, especially if the amount of injury energy is high. Almost all patients in the present study who sustained a facial fracture had an accompanying intracranial injury. Such combination injuries are generally more complex and can increase the duration of treatment and the number of necessary procedures^13,14^. However, no corresponding increase was observed in indirect costs, suggesting that although initial treatment may be expensive, recovery from these injuries is not necessarily hindered.

Accumulation of indirect costs are mostly explained by different social benefits and as anticipated, these were the highest for working age patients, most likely because of the paid sick leave they receive following an injury. Furthermore, indirect costs were higher in patients with a higher level of education, which can be explained by a higher sick leave allowance due to a higher salary. Lower ASA class was associated with higher indirect costs, most likely because working age patients are generally healthier than older patients. Fortunately, the difference in the costs between the 1st and 2nd year suggests that most these patients recover well from their injuries and the most expensive treatments take place soon after the injury. In Achit's study, most patients who were injured in a traffic accident recovered within 2 years, after which treatment costs were no longer incurred. In contrast, the same study reported that some patients sustained long-term disabilities, resulting in costs that will be incurred for many years^3,15^. Those patients had greater severity of injuries, and it may be assumed that some of the patients in our study will also have long-term or even permanent disability. Over time, these long-term or permanent disabilities will probably increase the amount of indirect costs in the future, but the monitoring period of our research material does not cover this.

A comparison with the costs reported in previous studies is challenging due to the different inclusion criteria used. Most of the existing literature consist of mainly mildly injured patients. In some studies, a description of injury severity or scores have not been reported or the definition of serious injury is different. For example, in a recent Danish study, Olesen et al. reported higher costs in unprotected patients, such as motorcyclists or pedestrians^16^, whereas we did not observe higher costs for injured pedestrians in our study. This may be explained by the different inclusion criteria used between studies, for example, when the inclusion criterion is a high injury score (NISS > 15), the influence of the injury mechanism probably becomes less important. Also, the social benefits vary among countries and thus comparison of indirect costs between countries can be misleading.

Interestingly, despite our data only includes patients with the most difficult injuries it can be argued that the average 2-year treatment cost of severe injury is quite reasonable. For example, in comparison, in Finland the cost of hip replacement surgery in 2020 was 6,124 euros^17^. However, the average cost does not emphasize the most expensive injury patients whose total costs may tenfold exceed the average costs. Therefore, studies are needed to further identify factors for these highest costs.

This study is susceptible to selection bias because it includes only those patients who survived long enough to be admitted to the ICU/HDU. Therefore, we do not have information on the costs that were generated by the on-site and emergency room treatment of those patients who died before admitting to ICU/HDU. It should also be noted that material costs, such as damage to property and vehicles, etc., was not included in this study.

The strength of this study is in the broad coverage of the injury as well as cost data. First, according to Finnish law, all motorized vehicles must be insured. Second, all costs resulting from traffic injuries are paid for from these insurances (only gross negligence limits liability). Third, insurance companies must provide all information on the insurance costs paid to the Finnish Motor Insurers’ Centre. Finally, all insurance companies must be part of the Finnish Motor Insurers’ Centre. Therefore, the Finnish Motor Insurers’ Centre is a reliable source for data on the costs of all traffic injuries in Finland. In addition, we were able to combine the cost data with the individual medical records of patients and data from Statistics Finland.

## Conclusion

The largest proportion of the costs related to severe traffic injuries originate from direct treatment costs, such as hospital treatment, rehabilitation, and medicine costs. The cost variation among severely injured trauma patients is large, ranging from a few thousand euros to half a million euros; however, the major proportion of costs is generated by a small number of high-cost patients. Combined, this makes planning of resource use challenging and calls for further studies to further identify factors for highest costs. Treatment costs seem to increase with the severity of the injury, particularly when injury affects lower extremities or the face. Indirect costs were higher in working age patients and in patients with a higher level of education.

## Data Availability

The data that support the findings of this study are not openly available due to privacy restrictions and are available from the corresponding author (AR) upon reasonable request.
